# Advantages of statin usage in preventing fractures for men over 50 in the United States: National Health and Nutrition Examination Survey

**DOI:** 10.1371/journal.pone.0313583

**Published:** 2024-11-25

**Authors:** Xiaona Sun, Xiaoxiao Liu, Chenyi Wang, Yushuang Luo, Xinyi Li, Lijuan Yan, Yaling Wang, Kaifa Wang, Qiang Li

**Affiliations:** 1 School of Mathematics and Statistics, Southwest University, Chongqing, China; 2 Department of Hypertension and Endocrinology, Center for Hypertension and Metabolic Diseases, Chongqing Institute of Hypertension, Daping Hospital, Army Medical University, Chongqing, China; 3 Department of Nephrology, First Medical Center of Chinese PLA General Hospital, Nephrology Institute of the Chinese People’s Liberation Army, National Key Laboratory of Kidney Diseases, National Clinical Research Center for Kidney Diseases, Beijing Key Laboratory of Kidney Disease Research, Beijing, China; 4 Department of Urology Surgery, Daping Hospital, Army Medical University, Chongqing, China; 5 Department of Nursing, Daping Hospital, Army Medical University, Chongqing, China; HT Ong Heart Clinic, MALAYSIA

## Abstract

**Objectives:**

The relationship between statin treatment and fracture risk is still controversial, especially in in patients with cardiovascular diseases (CVDs). We aim to determine whether statin therapy affects the occurrence of fractures in the general US population and in patients with CVDs.

**Methods:**

Epidemiological data of this cross-sectional study were extracted from the National Health and Nutrition Examination Survey (NHANES, 2001–2020, n = 9,893). Statins records and fracture information were obtained from the questionnaires. Weighted logistic regressions were performed to explore the associations between statin and the risk of fracture.

**Results:**

Statin use was found to be associated with reduced risk of fracture mainly in male individuals aged over 50 years old and taking medications for less than 3 years, after adjusted for confounders including supplements of calcium and vitamin D. The protective effects were only found in subjects taking atorvastatin and rosuvastatin. We found null mediation role of LDL-C and 25(OH)D in such effects. Statin was found to reduce fracture risk in patients with cardiovascular diseases (CVDs, OR: 0.4366, 95%CI: 0.2664 to 0.7154, *P* = 0.0014), and in patients without diabetes (OR: 0.3632, 95%CI: 0.1712 to 0.7704, *P* = 0.0091).

**Conclusions:**

Statin showed advantages in reducing risk of fracture in male individuals aged over 50 years old and taking medications for less than 3 years. More research is needed to determine the impact of gender variations, medication duration, and diabetes.

## Introduction

The 3-hydroxy-3-methylglutaryl coenzyme A reductase (HMGCR) inhibitors (statins) are widely used for the primary prevention of cardiovascular diseases (CVDs) [1]. In addition to their well-known cholesterol-lowering properties, other advantageous pleiotropic effects of statins have been noticed, of particularly is their effect on bone metabolism. The earliest report had been demonstrated statins to promote bone growth associated with the upregulation of bone morphogenetic protein -2 (BMP-2) [2]. And thereafter statins were found to exhibit stimulation of osteoblast differentiation, suppression of osteoblast death, and inhibition of osteoclastogenesis [3]. Thus, statins are expected to have both cardioprotective and osteoprotective effects, and possibly used for prevention of osteoporosis [4].

However, observations from different studies remained inconclusive on the protective role of statins on osteoporosis and related fracture. Several observational studies have found the association of statins use with improved bone mineral density (BMD), as well as reduced risk of fractures [4–13]. However, some other observational studies and post hoc analysis of randomized clinical trials (RCTs) in patients with CVDs have not found consistent results, and in some cases, have even reported findings that contradict these results [14–23]. Furthermore, previous systemic reviews and meta-analyses also reported indefinite conclusions on the associations between statins usage and the risk of fracture, or with osteoporosis and BMD [12, 24–30]. Numerous factors were suggested contributing to the discrepancy among these studies, including study design, gender, ethnicity, statin dosage, length of treatment, and the specific statins used. However, the main comorbidities in the population using statins, such as coronary heart disease, hypertension, diabetes, and stroke, have not been analyzed as important influencing factors in previous studies. Current guidelines for the treatment of hypercholesterolemia in high-risk patients suffering from CVDs or diabetes strongly recommended cholesterol levels to be as low as possible. Thus, it is of great significance to elucidate the impact of long-term use of statins on the risk of fractures in patients with CVDs.

Due to the inconsistent findings of previous observational studies, we conducted a serial cross-sectional analysis of data from eight separate 2-year (2001–2010, 2013–2014, and 2017–2020) of the National Health and Nutrition Examination Surveys (NHANES) to determine the relationship between statins and risk of fracture in different CVDs. Mediation analysis was performed to figure out the mediating role of low-density lipoprotein cholesterol (LDL-C) and 25-hydroxyvitamin D (25(OH)D).

## Materials and methods

### Study participants

**Fig 1:**
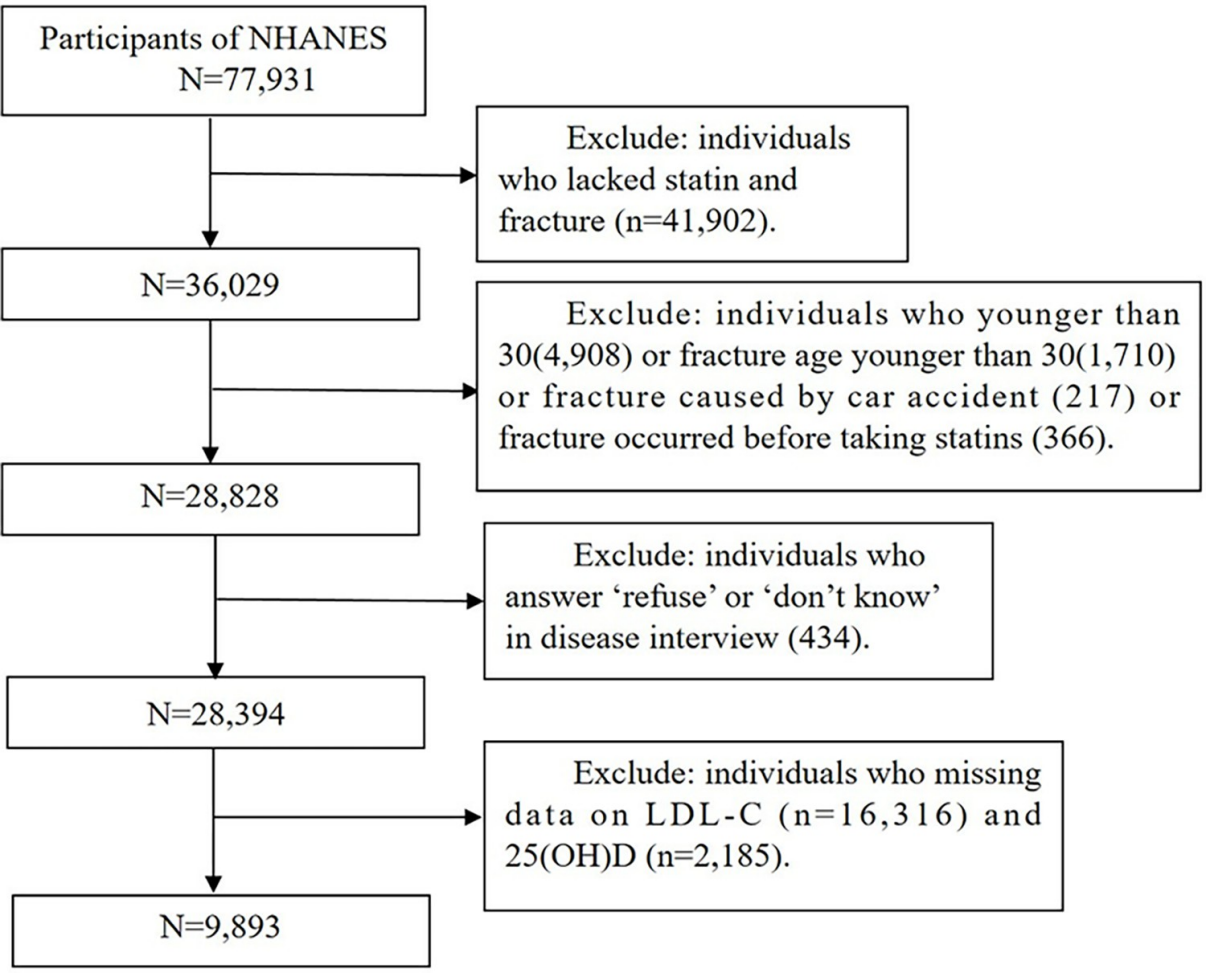
Flow chart of the screening process for the selection of eligible participants. LDL-C, low density lipoprotein cholesterol; 25(OH)D, 25-hydroxyvitamin D.

The data for this study comes from the NHANES (National Health and Nutrition Examination Survey) database, which is collected on a two-year cycle, and participants are required to provide informed consent. This study utilized NHANES data from various time periods: 2001–2010, 2013–2014, and 2017–2020. The years 2011–2012 and 2015–2016 were excluded from the analysis as they did not contain fracture records. In total, there were 77,931 records available for analysis. To ensure the accuracy of the study, firstly, any missing data related to fractures and statin use were eliminated, resulting in 36,029 participants. Secondly, in order to ensure as reliable as possible the effect of statins on fractures, excluding data from interviews with individuals under 30 years old(n = 4,908), those are younger than 30 years of age at the time of fracture (n = 1,710), those with fractures caused by car accidents(n = 217), and those with fractures occurring before statin medication(n = 366). Additionally, excluding participants who answered ‘refuse’ or ‘don’t know’ in disease interviews (n = 434). Last, individuals lacking data on LDL-C (n = 16,316) and 25(OH)D (n = 2,185) were also eliminated. And ultimately, a total of 9893 participants were included in the study (Fig 1). All methods are conducted in accordance with relevant guidelines and regulations. Ethics approval was obtained by the National Center for Health Statistics and informed consent was obtained for all participants. Ethical review and approval were waived for this study due to the use of publicly available data.

### Statins and bone fracture

Prescription statins records were obtained from the questionnaire data, which were administered by trained interviewers. The interviews took place in participants’ homes and used a computer-assisted personal interview (CAPI) system. During the household interviews, participants were asked whether they had taken any prescription medication within the past 30 days. Specifically, they were queried about seven statin drugs: Atorvastatin, Rosuvastatin, Simvastatin, Fluvastatin, Lovastatin, Pitavastatin and Pravastatin. Participants who responded "yes" were asked to present their medication containers of all products they had used to the interviewer. The interviewer recorded the full name of each reported drug into the computer. If no container was available, participants were asked to verbally report the name of the medications. Additionally, participants reported the duration of time they had been taking the prescribed medication, which was recorded in days, weeks, months, or years. For analysis purposes, all responses were converted to days using conversion factors of 7 days per week, 30.4 days per month, and 365 days per year. Information on self-reported hip fracture, wrist fracture, or spine fracture were obtained from the osteoporosis section of the questionnaire. The occurrence of a hip, wrist, or spine fracture was defined as having experienced a fracture.

### Clinical examination

Laboratory data was used to obtain measurements of serum 25(OH)D. For NHANES 2001–2006, a regression method was applied to convert the data to equivalent 25(OH)D measurements obtained from a standardized liquid chromatography-tandem mass spectrometry (LC-MS/MS) method. The standardized LC-MS/MS method was used in the year 2007–2010. In 2013–2014 and 2017–2018, the ultra-high performance liquid chromatography-tandem mass spectrometry (UHPLC-MS/MS) method and high-performance liquid chromatography-tandem mass spectrometry (HPLC-MS/MS) method were used, respectively. These methods were utilized to quantitatively determine the level of 25(OH)D3, epi-25(OH)D3, and 25(OH)D2. The sum of 25(OH)D2 and 25(OH)D3 represents the total 25(OH)D concentration. The measurements of BMD in the femur and spine were recorded for the years 2005–2020 using dual-energy X-ray absorptiometry (DXA) at the NHANES Mobile Examination Center.

### Covariates

The NHANES investigators collected demographic information from participants residing in the sampled area. In order to control the effects of confounding factors, the following covariates were identified: age (actual value), gender (male or female), race (Mexican American, Other Hispanic, Non-Hispanic White, Non-Hispanic Black, Other Race), education level (Less than 9th grade, 9th-11th grade, High school graduate/GED or equivalent, Some college or AA degree, College graduate or above), Ratio of family income to poverty (PIR), body mass index (BMI), high-density lipoprotein cholesterol (HDL-C), LDL-C, Total Cholesterol (TC), Triglyceride (TG), Aspartate Aminotransferase (AST), Alanine Aminotransferase (ALT), Serum Creatinine (SCR), Blood Urea Nitrogen (BUN), 25(OH)D, Glycohemoglobin (HbA1c), Smoking (not at all; some day; every day), Alcohol consumption (never: never, lightly: less than 2 drinks per day on average in the past year; moderately: less than or equal to 3 drinks per day on average in the past year; Severely: greater than 3 drinks per day on average in the past year), supplements of calcium and vitamin D. The PIR is calculated by dividing the household or individual income by the Department of Health and Human Services (DHHS) poverty guidelines for the year of the survey. Additionally, information on certain diseases was collected, including hypertension (yes: self-reported physician diagnosis or systolic blood pressure (SBP) ≥ 130 or diastolic blood pressure (DBP) ≥ 80; or no), diabetes (yes: self-reported physician diagnosis or HbA1c ≥6.5% or fasting blood glucose (FBG) ≥7.0 mmol/L or oral glucose tolerance test 2-hour blood glucose ≥11.1 mmol/L); or no), arthritis, congestive heart failure, coronary heart disease, angina, heart attack, stroke, thyroid problem and liver problem.

### Statistical analysis

For the epidemiological observational studies, numerical results are presented as mean ± SD, median with interquartile range, or n (%). Due to the large sample size and overly sensitive nature of the normality test, we employed graphical method in conjunction with skewness and kurtosis to determine whether the data conformed to a normal distribution. Sample data with an absolute value of kurtosis less than 10 and an absolute value of skewness less than 3 were considered to approximately normally distribute [31]. Continuous variables that followed normal distribution were described using weighted means and weighted standard deviations, and that did not follow normal distribution were described using weighted medians and weighted interquartile ranges. Categorical variables were described using frequencies and weighted percentages. Differences among normal continuous variables between two groups were analyzed using independent samples T-tests, differences among non-normal continuous variables between two groups were analyzed using Kruskal-Wallis tests, and differences among categorical variables were analyzed using Pearson’s chi-square tests.

The statistical analysis of this study was conducted in three steps. Firstly, we constructed four weighted logistic regression models with progressively increasing control variable to analyze the relationship between statins and fracture as well as the impact of gender disparities on the association between statins and fracture. Furthermore, we performed subgroup analyses for different age groups and examined the impact of statin use on fractures using weighted logistic regression models after controlled for all covariates. Secondly, the association between types or the cumulative number of days of statins medication and fractures was analyzed by weighted logistic regression modeling. Thirdly, mediation effect modeling [32]was used to explore whether LDL-C and 25(OH)D can mediate the association between statins and BMD or fractures. In this study, LDL-C or 25(OH)D was considered as the mediator variable, statins as the independent variable, and bone mineral density or fracture as the dependent variable. After constructing the mediator model, we tested the mediating effect using either the distribution of the product or the Bootstrap method [33, 34].

The study was statistically analyzed using R, version 4.3.2 (R Foundation for Statistical Computing) software, and a two-sided *P* < 0.05 was considered statistically significant. Considering the complex sampling design of the NHANES database, all analyses were adjusted for the survey design and weighting variables, which were created following the NHANES analysis guidelines. Due to the analysis data ending at record number 83,723, which belongs to the 2013–2014 cycle, only data from 6 cycles were involved. A new weight variable was created, that is the fasting subsample weight divided by the number of cycles (6). The sample data was weighted using the survey package in R, and all analyses were adjusted based on the survey design and weighted variables.

## Results

### General characteristics of participants

In total of 9,893 participants were included in this study (Table 1). Approximately 19% of the participants had been taking statin medications. Aside from the liver problems, these participants were more likely to be older, have a higher BMI, lower HDL-C, LDL-C and TC levels but higher TG levels, higher glycohemoglobin levels, and a higher chance of having conditions such as arthritis, diabetes, hypertension, heart diseases and stroke. A noteworthy discovery was that despite higher levels of 25(OH)D in the statin group compared to non-statin group, these participants exhibited lower femoral BMD. We observed that the fracture group had an older age and a higher likelihood of fractures in females compared to males. Participants in fracture group exhibited lower femoral and spinal BMD as expected. However, none of differences in 25(OH)D were observed in patients with and without fractures.

**Table 1:** Baseline characteristics of participants.

Characteristics	Statin use		P Value	Fracture		P Value
	Non (7985)	Yes (1908)		Non (9412)	Yes (481)	
Age, mean (SD)	50.32(13.63)	63.00(11.58)	<0.001	52.22(14.04)	59.53(14.41)	<0.001
Gender						
Male	3671(44.5)	995(51.5)	<0.001	4468 (46.0)	198(40.7)	0.074
Female	4314(55.5)	913(48.5)		4944 (54.0)	283(59.3)	
Race/Ethnicity						
Mexican American	1544(7.9)	214(3.5)	<0.001	1681(7.2)	77(5.2)	0.016
Other Hispanic	604(4.5)	113(2.3)		694(4.2)	23(3.0)	
Non-Hispanic White	3835(70.2)	1117(78.9)		4649(71.4)	303(79.4)	
Non-Hispanic Black	1560(11.7)	346(9.2)		1848(11.5)	58 (6.7)	
Other Race	442(5.6)	118(6.2)		540(5.7)	20(5.8)	
Education level						
Less than 9th grade	1170(7.2)	256(7.1)	0.089	1347(7.1)	79(8.9)	0.024
9-11th grade	1175(11.5)	299(11.3)		1397(11.4)	77(12.0)	
High school graduate/GED	1760(23.2)	474(27.2)		2112(23.7)	122(28.2)	
Some college or AA degree	2115(29.3)	466(26.7)		2455(28.7)	126(30.3)	
College graduate or above	1765(28.9)	413(27.8)		2101(29.1)	77(20.7)	
PIR						
<130%	2054(17.7)	444(16.1)	0.413	2345(17.1)	153(24.0)	0.001
130%-349%	2846(35.9)	711(36.9)		3385(36.0)	172(38.7)	
≥350%	2505(46.4)	625(47.0)		3017(46.9)	113(37.3)	
BMI (kg/m^2^), mean (SD)	28.61(6.60)	30.01(6.62)	<0.001	28.91(6.65)	27.95(6.04)	0.013
HDL-c (mg/dL), mean (SD)	55.37 (16.72)	52.75 (14.33)	<0.001	54.89 (16.33)	55.11 (16.87)	0.852
LDL-c (mg/dL), mean (SD)	123.56(34.60)	98.97(32.16)	<0.001	119.04(35.43)	122.30(35.70)	0.140
TC (mg/dL), mean (SD)	204.06(39.02)	180.00(38.26)	<0.001	199.57(39.86)	204.22(41.63)	0.077
TG (mg/dL), median (IQR)	108.0[77.0,156.0]	124.0[89.0,179.0]	<0.001	111.0[78.0,161.0]	114.0[82.0,72.0]	0.201
ALT (U/L), median (IQR)	21.0[16.0,28.0]	22.0[18.0,28.0]	0.019	21.0[17.0,28.0]	20.0[17.0,28.0]	0.39
AST (U/L), median (IQR)	22.0[19.0,27.0]	24.0[20.0,28.0]	<0.001	23.0[19.0,27.0]	23.0[20.0,28.0]	0.069
BUN (mg/dL), median (IQR)	12.0[10.0,15.0]	15.0[12.0,18.9]	<0.001	13.0[10.0,16.0]	13.0[11.0,16.0]	0.105
SCR (mg/dL), median (IQR)	0.84[0.7,1.0]	0.9[0.8,1.1]	<0.001	0.87[0.7,1.0]	0.82[0.8,1.0]	0.744
25(OH)D (nmol/L), mean (SD)	64.99(24.98)	69.67(26.31)	<0.001	65.71(25.30)	68.09(24.88)	0.065
HbA1c (%), median [IQR]	5.4[5.2,5.7]	5.7 [5.5,6.3]	<0.001	5.4[5.2,5.7]	5.5[5.2,5.8]	0.174
FemurBMD(gm/cm2),mean(SD)	0.82(0.14)	0.78(0.15)	<0.001	0.81(0.14)	0.74(0.15)	<0.001
SpineBMD(gm/cm2),mean (SD)	1.03(0.15)	1.03(0.16)	0.773	1.03(0.15)	0.97(0.15)	<0.001
Alcoholic use						
Never	2259(29.4)	597(34.2)	<0.001	2691(29.8)	165(39.5)	0.012
Lightly	1465(25.2)	412(30.2)		1795(26.1)	82(24.1)	
Moderately	1843(34.1)	357(29.9)		2125(33.7)	75(26.3)	
Severely	718(11.3)	79(5.7)		763(10.3)	34(10.1)	
Smoking						
Not at all	6342(78.8)	1638(85.9)	<0.001	7617(80.4)	363(72.1)	0.001
Some day	241(2.7)	44(1.9)		275(2.6)	10(2.0)	
Every day	1397(18.5)	226(12.3)		1515(17.0)	108(25.9)	
Arthritis						
Yes	2266(26.8)	940(46.5)	<0.001	2942(29.2)	264(51.7)	<0.001
No	5719(73.2)	968(53.5)		6470(70.8)	217(48.3)	
Diabetes						
Yes	1745(6.1)	924(26.9)	<0.001	2554(9.7)	115(10.2)	0.905
No	4805(93.9)	755(73.1)		5279(90.3)	281(89.8)	
Hypertension						
Yes	4364(29.8)	1543(66.0)	<0.001	5572(35.8)	335(45.3)	0.001
No	3620(70.2)	365(34.0)		3839(64.2)	146(54.7)	
Congestive heart failure (%)						
Yes	188(1.8)	172(7.0)	<0.001	334(2.6)	26(5.0)	0.006
No	7797(98.2)	1736(93.0)		9078(97.4)	455(95.0)	
Coronary Heart Disease (%)						
Yes	163(1.5)	320(16.1)	<0.001	454(4.0)	29(4.7)	0.432
No	7822(98.5)	1588(83.9)		8958(96.0)	452(95.3)	
Angina (%)						
Yes	144(1.2)	182(9.4)	<0.001	299(2.6)	27(3.5)	0.155
No	7841(98.8)	1726(90.6)		9113(97.4)	454(96.5)	
Heart attack (%)						
Yes	225(2.1)	280(13.4)	<0.001	475(4.0)	30(5.3)	0.23
No	7760(97.9)	1628(86.6)		8937(96.0)	451(94.7)	
Stroke (%)						
Yes	243(2.3)	195(8.4)	<0.001	405(3.3)	33(5.5)	0.024
No	7742(97.7)	1713(91.6)		9007(96.7)	448(94.5)	
Thyroid problem (%)						
Yes	546(7.3)	260(14.6)	<0.001	755(8.4)	51(11.1)	0.091
No	7388(92.7)	1640(85.4)		8603(91.6)	425(88.9)	
Liver problem (%)						
Yes	147(1.8)	30(1.5)	0.478	162(1.6)	15(4.0)	0.010
No	7783(98.2)	1868(98.5)		9187(98.4)	464(96.0)	

SI conversion factors: To convert cholesterol to mmol/L, multiply values by 0.0259. PIR, ratio of family income to poverty; BMI, Body mass index; HDL-c, high-density lipoprotein cholesterol; LDL-c, low-density lipoprotein cholesterol; TC, total cholesterol; TG, triglycerides; ALT, alanine transaminase; AST, aspartate transaminase; BUN, blood urea nitrogen; SCR, serum creatinine; 25(OH)D, 25-hydroxyvitamin D; HbA1c, glycated hemoglobin; BMD, bone mineral density.

### Comparison of the risk of bone fracture between patients with and without statin treatment

We developed several weighted multifactorial logistic regression models to explore the effect of statins treatment on fracture risk by continuously adding control variables. In present analysis, bone fracture was uncommon in patients treated with statin compared to non-statin group (OR: 0.4538, 95% CI: 0.2705 to 0.7612, *P* = 0.0034), after adjusted for all baseline influencing factors (Table 2). In a sex-specific analysis, the protective effect of statin was overrepresented in male (OR: 0.2448, 95% CI: 0.0955 to 0.6272, *P* = 0.0040) and marginal significantly in female participants (OR: 0.5536, 95% CI: 0.3120 to 0.9823, *P* = 0.0435) (Table 2). In the age-stratified population analysis, after categorizing the participants into two age groups- 30–50 years, and greater than 50 years, the association between statin treatment and a decreased likelihood of fracture risk was only observed in participants aged over 50 years old (OR_>50_: 0.4755, 95% CI: 0.2894 to 0.7812, *P* = 0.0040, S1 Table). Similarly, we only found the protective effect of statin usage on fracture risk in male participants over the age of 50 (OR_>50_: 0.2061, 95% CI: 0.0786 to 0.5402, *P* = 0.0017, S1 Table), while totally none protective role in female participants (S1 Table).

**Table 2:** Association between facture risk and statin treatment.

Model	Statin use			
	Ref	Beta	OR (95%CI)	P Value
Participants = Overall (7134)				
Crude	1	-0.3933	0.6748(0.4240, 1.0742)	0.0963
Model1	1	-0.8155	0.4424(0.2649, 0.7388)	0.0022
Model2	1	-0.7937	0.4521(0.2695, 0.7587)	0.0032
Model3	1	-0.7901	0.4538(0.2705, 0.7612)	0.0034
Participants = male (3211)				
Crude	1	-1.2238	0.2941(0.1273, 0.6797)	0.0047
Model1	1	-1.5258	0.2174(0.0881, 0.5366)	0.0012
Model2	1	-1.4072	0.2448(0.0935, 0.6410)	0.0048
Model3	1	-1.4075	0.2448(0.0955, 0.6272)	0.0040
Participants = female (3923)				
Crude	1	0.0346	1.0352(0.6234, 1.7191)	0.8925
Model1	1	-0.5370	0.5845(0.3293, 1.0376)	0.0663
Model2	1	-0.5843	0.5575(0.3151, 0.9865)	0.0449
Model3	1	-0.5913	0.5536(0.3120, 0.9823)	0.0435

No statins were taken as the baseline group. The analysis was conducted using a weighted logistic regression model. The crude model did not adjust for any covariates. Model 1 was adjusted for age, gender, race, education, PIR, and BMI. Model 2 was adjusted for all the factors in Model 1, as well as LDL-Cholesterol (1-SD), HDL-Cholesterol (1-SD), Total Cholesterol (1-SD), Triglyceride (1-SD), Aspartate Aminotransferase (AST) (1-SD), Alanine Aminotransferase (ALT) (1-SD), Serum Creatinine (1-SD), Blood Urea Nitrogen (1-SD), 25-hydroxyvitamin D (1-SD), and HbA1c (1-SD). Model 3 was adjusted for all the factors in model2 plus Alcoholic use, smoking status and supplements of calcium and vitamin D.

We further developed a mediation model to explore the relationship between statin usage and risk of fracture, with LDL-C and 25(OH)D as mediating variables. Unfortunately, we did not find a mediating effect (S2 Table and S1 Fig).

### Comparison of the different types, and treatment time of statins with the risk of fracture

Through the use of weighted multivariate logistic regression with no statins as the reference group, we discovered that patients who took atorvastatin (OR _atorvastatin_: 0.4619, 95% CI: 0.2376 to 0.8980, *P* < 0.05) and rosuvastatin (OR _Rosuvastatin_: 0.1054, 95% CI: 0.0199 to 0.5586, *P* < 0.05) were at a lower risk of suffering from fractures (S3 Table). Participants were then grouped into three categories based on their duration of statin usage: 0–30.4, 30.4–1095, and >1095 days. After adjusting for confounding factors and using the 0–30.4 days group as reference, statin was identified as a statistically significant protective factor for fracture in patients who took statin treatment for less than 1095 days (OR _30.4–1095_: 0.3119, 95% CI: 0.1487 to 0.6540, *P* = 0.0026). However, this protection disappears when taking statins exceeding 1095 days (OR _>1095_: 0.6353, 95%CI: 0.3278 to 1.2314, *P* = 0.1752, Table 3). Furthermore, sex-specific analysis also found similar results (OR _male:30.4–1095_: 0.1229, 95% CI: 0.0372 to 0.4067, *P* = 0.0009; OR _female:30.4–1095_: 0.3987, 95% CI: 0.1782 to 0.8922, *P* = 0.0261, Table 3).

**Table 3:** Relationship between the number of days of statins and fracture.

Fracture	participants = overall (6672)		participants = male (3030)		participants = female (3642)	
Medication days	OR (95%CI)	P Value	OR (95%CI)	P Value	OR (95%CI)	P Value
0–30.4(5398/2388/3027)	1		1		1	
30.4-1095(633/305/328)	0.3119(0.1487, 0.6540)	0.0026	0.1229(0.0372, 0.4067)	0.0009	0.3987(0.1782, 0.8922)	0.0261
>1095(641/337/287)	0.6353(0.3278, 1.2314)	0.1752	0.4311(0.1317, 1.4106)	0.1607	0.7413(0.3452, 1.5919)	0.4363

The analysis was conducted using a weighted logistic regression model and adjusted age, gender, race, education, PIR, BMI, LDL-Cholesterol (1-SD), Total Cholesterol (1-SD), HDL-Cholesterol (1-SD), Triglyceride (1-SD), Aspartate Aminotransferase (AST) (1-SD), Alanine Aminotransferase (ALT) (1-SD), Serum Creatinine (1-SD), Blood Urea Nitrogen (1-SD), 25-hydroxyvitamin D (1-SD), and HbA1c (1-SD), Alcoholic use, smoking status, supplements of calcium and vitamin D.

### Association of statin use and fracture risk in patients with CVDs

Hypertension, arthritis, and diabetes are the three most common comorbidities in the database. We then investigated how statin therapy affected fracture risk across a range of comorbidities (Table 4). Statin was found to play a protective role against fracture risk in patients with arthritis (OR: 0.4910, 95%CI: 0.2606 to 0.9251, *P* = 0.0284), hypertension (OR: 0.3960, 95%CI: 0.2369 to 0.6619, *P* = 0.0006), and in patients without diabetes (OR: 0.3632, 95%CI: 0.1712 to 0.7704, *P* = 0.0091) or arthritis (OR: 0.4034, 95%CI: 0.1816 to 0.8963, *P* = 0.0265). Only marginal significance was observed in patients with diabetes (*P* = 0.0435), and the benefits of statins in lowering fracture risk were vanished in hypertensive patients with diabetes (*P* = 0.1341). Moreover, individuals with conventional CV illnesses, such as hypertension, stroke, and congestive heart failure, also showed similar protective effects (OR: 0.4366, 95%CI: 0.2664 to 0.7154, *P* = 0.0014). By using multiple confounder adjustment in linear regression analysis, we discovered that an increase in spine BMD was associated with decrease in LDL in patients with diabetes (β: -10.9319, 95%CI: -19.0744 to -2.7893, *P* = 0.0101) and without hypertension (β: -7.9945, 95%CI: -13.2323 to -2.7568, *P* = 0.0039), and this correlation persisted with femoral BMD in patients without hypertension (S4 Table).

**Table 4:** Relationship between statin and fracture in different disease populations.

Diseases	Beta	OR (95%CI)	P Value
Diabetes (5938)			
Yes (1829)	-0.8041	0.4475(0.2052, 0.9761)	0.0435
No (4109)	-0.1013	0.3632(0.1712, 0.7704)	0.0091
Hypertension (7134)			
Yes (4225)	-0.9264	0.3960(0.2369, 0.6619)	0.0006
No (2909)	-0.3309	0.7183(0.2581, 1.9991)	0.5201
Arthritis (7134)			
Yes (2255)	-0.7112	0.4910(0.2606, 0.9251)	0.0284
No (4879)	-0.9077	0.4034(0.1816, 0.8963)	0.0265
Hypertension/Stroke/Congestive Heart Failure (7134)			
Yes (4294)	-0.8288	0.4366(0.2664, 0.7154)	0.0014
No (2840)	-0.4016	0.6692(0.2038, 2.1977)	0.5017
Hypertension & Diabetes (5938)			
Yes (1349)	-0.6174	0.5393(0.2391, 1.2165)	0.1341
No (1849)	-0.0977	0.9069(0.2860, 2.8759)	0.8661

The analysis was conducted using a weighted logistic regression model and adjusted age, gender, race, education, PIR, BMI, LDL-Cholesterol (1-SD), Total Cholesterol (1-SD), HDL-Cholesterol(1-SD), Triglyceride (1-SD), Aspartate Aminotransferase (AST) (1-SD), Alanine Aminotransferase (ALT) (1-SD), Serum Creatinine (1-SD), Blood Urea Nitrogen (1-SD), 25-hydroxyvitamin D (1-SD), and HbA1c (1-SD), Alcoholic use, smoking status, supplements of calcium and vitamin D.

## Discussion

The current study examined the association between the use of statins and the risk of fractures by epidemiological data from the NHANSE. We found that statin could reduce risk of fracture mainly in male individuals aged over 50 years old and taking medications for less than 3 years, and such protective effects were only found in atorvastatin and rosuvastatin. Statin was found to reduce fracture risk in patients with CVD, including hypertension, stroke, and congestive heart failure, and in patients without diabetes.

### Potential mechanism

Cholesterol homeostasis is important in regulating the proliferation and stimulation of osteoblasts and osteoclasts, as well as bone metabolism. Golgi-resident site-1 protease (S1P), LXR (α, β), LDL receptor (LDLR), ATP-binding cassette transporter A1 (ABCA1), and lysosomal acid lipase (LAL) have been suggested involving in the relationship between cholesterol metabolism and osteoporosis [3, 35]. Oxidized LDL (oxLDL), which is critical in the initiation and progression of atherosclerosis, had also been reported to be linked to osteoclastogenesis, bone resorption, and osteoblast demineralization through elevating levels of the receptor activator of NF-kappaB ligand (RANKL), or inhibiting phosphate signaling and phosphate-induced mineralization [36, 37]. Furthermore, recent study found that osteoblast demineralization could be induced by oxidized HDL via the inflammatory pathway [38].

Numerous mechanisms had been proposed potential beneficial effects of statins on bone metabolism. Statins were suggested to be contributed to the bone formation through multiple ways, including enhanced BMP-2 expression [2], inhibited osteoclast activity through reducing the synthesis of FPP and GGPP, blocking the Ras/ERK pathway, and activating p38MAPK [39, 40], inhibited osteoblastic apoptosis through the TGFβ/Smad3 signaling pathway [41, 42], decreased osteoclastogenesis by regulating the estrogen receptor or osteoprotegerin/RANKL/RANK pathway [43–45], increased 25-hydroxy-vitamin D concentrations [46], and fostered osteoblast activity [47, 48].

In contrary, there is also concern on the potential deleterious consequences of statins impacts on sex hormone levels such as testosterone or estrogen [49]. since the endogenous synthesis of cholesterol was the main substrate for the synthesis of sex hormones. Particularly, in the postmenopausal state, oestrogen is crucial for the maintenance of BMD [50]. Both in mice models and cell lines studies have found that statins could reduce plasma levels of testosterone, oestradiol and progesterone [51, 52]. Furthermore, decreased BMD by various statins were also discovered in rodents’ study [53].

### Comparison with other studies

Osteoporosis was more common in postmenopausal women, and in men aged 50 years or older [54, 55]. In the young, fractures occur more frequently in males, whereas from the age of 50 years onwards, fractures in females predominate, and the rates become approximately twice those in men [56, 57]. Several population-based cohort studies in Asia with subjects aged over 50 years, found that statin use was associated with a decreased risk of new-onset osteoporosis or facture in both genders [48, 58, 59]. This is supported by earlier researches on populations in Europe, America, and even elderly patients [6–8], and also in postmenopausal women [5, 9]. Our study’s general population analysis was consisted with earlier researches showing the beneficial effects of statin medication on lowering the risk of fracture. On the other hand, a number of randomized clinical trials, such as the JUPITER, Scandinavian Simvastatin Survival Study (4S), Long Term Intervention with Pravastatin in Ischemic Disease (LIPID), and Heart Protection Study (HPS) studies, discovered that statins for CVDs did not reduce the risk of fracture in individuals of a comparable age [21, 22, 60, 61]. Contradictory result was observed in our study. We also found decreased risk of fracture in patients with hypertension, stroke, and congestive heart failure who used statins. However, such benefits were not found in patients with diabetes, but in patients without diabetes. Results from the Women’s Health Initiative Observational Study also found none beneficial effects of statins on improving fracture risk [15]. A retrospective examination of Austrian population revealed that statin use was linked to an overrepresentation of osteoporosis diagnoses in both sexes, particularly in the age-class of 40–50 years [17]. Furthermore, another case-control study found that statin use was related to an increased rate of osteoporosis in the ≥60-year-old female group [62]. In contrast, we found decreased fracture risk in male participants aged over 50 years old, while none role of statin uses on risk of fracture in female subjects. Likewise, statins did not lower the incidence of fracture in the research for the treatment of osteoporosis that involved mostly female participants [63]. Noteworthy, we also found that statin was associated with reduced fracture risk in patients with CVDs. However, we did not discover any such preventive benefits of statins on the risk of fractures in patients with diabetes. Given the increased risk of fractures and lipid abnormalities associated with diabetes, this finding may help to explain why the fracture-protective benefit of statins was not seen in earlier subgroup analyses in RCT studies. This impact may be related to the high blood sugar levels observed in these patients.

Additionally, there were debatable topics regarding the possible impacts on bone metabolism of various statin kinds, dosages, and durations. Longer statin duration, higher cumulative dose, or higher statin intensity may be linked to a lower risk of significant osteoporotic fracture, according to certain observational studies [48, 58, 59]. In contrast, research by *Leutner M* and colleagues demonstrated that osteoporosis diagnosis in statin-treated patients was dose-dependent, with a higher incidence of osteoporosis in those on high-dose statin therapy [17]. And they also showed that the dosage-dependent relationship between statin use and osteoporosis risk would not be confounded by comorbidities such as CVDs, overweight and obesity, stroke, diabetes, *etc*. Two additional studies found no associations between the duration of statin use and the risk of osteoporosis or fracture [15, 62]. Similarly, we discovered that statin therapy was linked to a lower incidence of fracture, but only in those who had treatment for fewer than three years, after which the beneficial benefits vanished. Statins are categorized as lipophilic (atorvastatin, simvastatin, pitavastatin, and lovastatin) and relatively hydrophilic (fluvastatin, pravastatin and rosuvastatin) based on their intrinsic polar properties. In our study, atorvastatin and rosuvastatin were found to be related with lower risk of suffering from fractures and rosuvastatin reduces more risk of fractures. This difference in efficacy could potentially be attributed to variances in their polarity and bone bioavailability. Studies have shown that lipophilic statins, but not hydrophilic statins, were associated with an increased rate of osteoporosis in the women aged over 60 years [62]. The high absorption rate of lipophilic statins could heighten the impact of estrogen deprivation in elderly women [62]. While hydrophilic statins have been reported to exhibit a decreased absorption rate and dependency on the cytochrome P450 enzyme, resulting in fewer adverse effects than lipophilic statins [64]. Furthermore, it was recently reported that patients on lipophilic statins had statistically lower BMD than females on hydrophilic statins, and the BMD decreased in a dose-dependent of statin [65]. However, it has also been found that lipophilic statins, instead of hydrophilic statins, have demonstrated superior outcomes in terms of osteoporotic fractures [25, 66], lipophilic statins have been found to enhance BMP-2 expression, which further promotes osteoblast differentiation.

Inconsistent outcomes regarding the impact of statins on fracture were also observed across different fracture sites. Traditionally, long bone fractures are the most common type of fracture seen in the young (as a result of substantial trauma), while the forearm, hip and vertebrae are the sites most susceptible to fracture in older individuals [55, 57, 67]. Several studies demonstrated protective role of statins on reducing hip and vertebral fractures compared to non-statins users [48, 59]. Nevertheless, meta-analysis of RCTs or observational studies found no association between statin use and fracture reduction, or only in hip fractures [27, 28]. Recent meta-analysis examined the effects of statin therapy in the elderly and discovered that the only fractures linked to statin use were those of the hip and lower extremities [26]. Notably, only less than one year duration of atorvastatin use was associated with a reduction in fracture risk [26]. The majority of vertebral fractures do not currently come to medical attention and thus remain undiagnosed [68]. However, vertebral fractures are strong risk factors for subsequent fracture at the spine and other skeletal sites [55].

Whether statins exert their effects on bone metabolism through regulating dyslipidemia is still controversial. Observational studies evaluating the association between lipids and BMD are inconsistent, with some studies showing no association, while others have reported either a negative or a positive effect [69]. Meta-analyses also did not overcome these discrepancies. Chen YY *et al*. compared the lipid profile in postmenopausal women found that HDL-C and TC concentrations were higher in the osteoporosis compared with the normal BMD group [70]. While another meta-analysis performed in patients with osteopenia or osteoporosis, only found HDL-C was elevated in patients with osteoporosis [71]. Besides, there was one newly cohort study suggested that higher levels of HDL-C are associated with an increased fracture risk in healthy older adults [72]. In our study, we did not find significant differences in lipid profiles between individuals with and without fracture at baseline. Furthermore, none mediating effects of either LDL and 25(OH)D were found between statins usage and fracture risk in mediation model analysis. Noteworthy, individuals with fracture had lower femur and spine BMD as expected, while decreased femur BMD was also found in subjects taking statins.

### Strengths and limitations of this study

This study has several important strengths. First, our study data was extracted from NHANES 2001–2020, which is a timely and of high-quality national population-based survey with large sample size. Second, we have effectively corrected the impact of trauma, medications (calcium and vitamin D supplements), and other confounding factors on the occurrence of fractures, thus further enhancing the reliability of the results. Third, we further explored the impact of statin use on the occurrence of fractures in patients with risk of cardiovascular events such as hypertension and diabetes. Thus, it has certain clinical guidance value in the use of statins in patients with comorbidities.

The current study has some shortcomings that need to be addressed. (1) NHANES was still cross-sectional design, which failed to provide longitudinal follow-up data and unable to establish causal relationships. A prospective study on the effect of statin use on osteoporosis is warranted. (2) NHANES is a nationwide survey, but its results mainly reflect the health status of the American population, so the universality of the research results for other countries or regions is still uncertain. (3) self-reported comorbidities were present, and 18,935 people were omitted due to incomplete data on any of the primary variables, potentially influencing the outcomes. (4) Although we have adjusted for majority of confounding factors in the model, there are still confounding factors that cannot be ruled out due to data limitations, such as physical activity, dietary factors, muscle loss, statin dosage and intensity.

## Conclusions

In summary, current study provided evidence that statin use was associated with reduced risk of fracture in patients with CVDs, mainly in male individuals and within a certain period of treatment. Patients undergoing long-term lipid-lowering therapy, especially female patients or patients with diabetes, require further clinical observation to determine the protective effects.

## Supporting information

S1 Table Age-specific analysis of association between facture risk and statin treatment. (DOCX)

S2 Table The mediating effect of LDL-C on the association between statin and femoral or spine BMD. (DOCX)

S3 Table Effect of different statin types on fractures. (DOCX)

S4 Table Association between LDL-C and BMD in different disease populations. (DOCX)

S1 Fig Mediation effect to LDL-C(A) or 25(OH)D(B)on the relationship between statin and fracture. Adjusted for age, gender, race, education, PIR, BMI, Total Cholesterol (1-SD), HDL- Cholesterol (1-SD), Triglyceride (1-SD), Aspartate Aminotransferase (AST), Alanine Aminotransferase (ALT) (1-SD), Serum Creatinine (1-SD), Blood Urea Nitrogen (1-SD), and HbA1c (1-SD), smoking and drinking status, supplements of calcium and vitamin D; CI, confidence interval.
(TIF)
