# Supplementary material for: Advantages of statin usage in preventing fractures for men over 50 in the United States: National Health and Nutrition Examination Survey
Source: PLoS One. 2024 Nov 25;19(11):e0313583. doi: 10.1371/journal.pone.0313583 (PMC11588256; doi:10.1371/journal.pone.0313583)
Supplement: S1 Table — (DOCX) [file pone.0313583.s001.docx]

**S1 Table: Age-specific analysis of association between facture risk and statin treatment.**

| **Age group** | **Statin use** | | | |
| --- | --- | --- | --- | --- |
|  | **Ref** | **Beta** | **OR (95%CI)** | **P Value** |
| **Participants=Overall(N=7134)** | | | | |
| 30-50(2840) | 1 | 0.1007 | 1.1060(0.2909, 4.2051) | 0.8806 |
| >50(4294) | 1 | -0.7434 | 0.4755(0.2894, 0.7812) | 0.0040 |
| **Participants=male(N=3211)** | | | | |
| 30-50(1251) | 1 | 0.0015 | 1.0015(0.1242, 8.0777) | 0.9989 |
| >50(1960) | 1 | -1.5793 | 0.2061(0.0786, 0.5402) | 0.0017 |
| **Participants=female(N=3923)** | | | | |
| 30-50(1589) | 1 | -0.1480 | 0.8624(0.0957, 7.7741) | 0.8933 |
| >50(2334) | 1 | -0.4927 | 0.6110(0.3561, 1.0484) | 0.0730 |

Abbreviation: No statins were taken as the baseline group. The analysis was conducted using a weighted logistic regression model and adjusted age, gender, race, education, PIR, BMI, LDL-Cholesterol (1-SD), HDL-Cholesterol (1-SD), Total Cholesterol (1-SD), Triglyceride (1-SD), Aspartate Aminotransferase (AST) (1-SD), Alanine Aminotransferase (ALT) (1-SD), Serum Creatinine (1-SD), Blood Urea Nitrogen (1-SD), 25-hydroxyvitamin D (1-SD), and HbA1c (1-SD), Alcoholic use, smoking status, supplements of calcium and vitamin D.
