# Supplementary material for: Advantages of statin usage in preventing fractures for men over 50 in the United States: National Health and Nutrition Examination Survey
Source: PLoS One. 2024 Nov 25;19(11):e0313583. doi: 10.1371/journal.pone.0313583 (PMC11588256; doi:10.1371/journal.pone.0313583)
Supplement: S2 Table — (DOCX) [file pone.0313583.s002.docx]

**S2 Table: The mediating effect of LDL-C on the association between statin and femoral or spine BMD.**

| **Mediation analysis** | **Statin-LDL-C-Femoral BMD** | | **Statin-LDL-C-Spine BMD** | |
| --- | --- | --- | --- | --- |
|  | **Beta(95%CI)** | **P Value** | **Beta(95%CI)** | **P Value** |
| Total effect | -0.0465(-0.1471, 0.0542) | 0.3532 | -0.0143(-0.1376, 0.1089) | 0.8142 |
| Direct effect | -0.0469(-0.1476, 0.0537) | 0.3485 | -0.0105(-0.1327, 0.1117) | 0.8620 |
| Path a | -0.0001(-0.0010, 0.0008) | 0.8039 | 1.46e-05(-0.00112, -0.00115) | 0.9793 |
| Path b | 0.0811(0.0249, 0.1871) | 0.1288 | -0.0458(-0.1622, 0.0705) | 0.4279 |
| Indirect effect | -3.15e-05(-8.16e-05, 5.75e-05) | 0.8080 | 2.82e-06(-5.80e-05, 5.93e-05) | 0.9760 |

Abbreviation: The analysis adjusted age, gender, race, education, PIR, BMI, HDL-Cholesterol(1-SD), Total Cholesterol (1-SD), Triglyceride (1-SD), Aspartate Aminotransferase (AST) (1-SD), Alanine Aminotransferase (ALT) (1-SD), Serum Creatinine (1-SD), Blood Urea Nitrogen (1-SD), 25-hydroxyvitamin D (1-SD), and HbA1c (1-SD), Alcoholic use, smoking status, supplements of calcium and vitamin D.
