# Supplementary material for: Advantages of statin usage in preventing fractures for men over 50 in the United States: National Health and Nutrition Examination Survey
Source: PLoS One. 2024 Nov 25;19(11):e0313583. doi: 10.1371/journal.pone.0313583 (PMC11588256; doi:10.1371/journal.pone.0313583)
Supplement: S3 Table — (DOCX) [file pone.0313583.s003.docx]

**S3 Table: Effect of different statin types on fractures.**

|  | **No** | **Atorvastatin (491)** | **Rosuvastatin (96)** | **Simvastatin (499)** | **Lovastatin (114)** | **Pravastatin (130)** |
| --- | --- | --- | --- | --- | --- | --- |
| Crude | 1 | 0.6058(0.3222, 1.1391) | 0.1373(0.0245, 0.7703) * | 0.8339(0.4294, 1.6191) | 0.9929(0.3430, 2.8745) | 0.6420(0.1195, 3.4489) |
| Model1 | 1 | 0.4432(0.2288, 0.8583) * | 0.1042(0.0184, 0.5907) * | 0.5769(0.2818, 1.1810) | 0.6730(0.2213, 2.0460) | 0.4340(0.0783, 2.4059) |
| Model2 | 1 | 0.4466(0.2307, 0.8647) * | 0.1039(0.0196, 0.5502) * | 0.5803(0.2741, 1.2283) | 0.7029(0.2417, 2.0435) | 0.4171(0.0774, 2.2471) |
| Model3 | 1 | 0.4619(0.2376, 0.8980) * | 0.1054(0.0199, 0.5586) * | 0.5899(0.2769, 1.2570) | 0.6727(0.2335, 1.9377) | 0.3938(0.0736, 2.1049) |

Abbreviation: No statins were taken as the baseline group. *, represents *P* < 0.05. The analysis was conducted using a weighted logistic regression model. The crude model did not adjust for any covariates. Model 1 was adjusted for age, gender, race, education, PIR, and BMI. Model 2 was adjusted for all the factors in Model 1, as well as LDL-Cholesterol (1-SD), HDL-Cholesterol (1-SD), Total Cholesterol (1-SD), Triglyceride (1-SD), Aspartate Aminotransferase (AST) (1-SD), Alanine Aminotransferase (ALT) (1-SD), Serum Creatinine (1-SD), Blood Urea Nitrogen (1-SD), 25-hydroxyvitamin D (1-SD), and HbA1c (1-SD). Model 3 was adjusted for all the factors in model2 plus Alcoholic use, smoking status and supplements of calcium and vitamin D.
