# Supplementary material for: Advantages of statin usage in preventing fractures for men over 50 in the United States: National Health and Nutrition Examination Survey
Source: PLoS One. 2024 Nov 25;19(11):e0313583. doi: 10.1371/journal.pone.0313583 (PMC11588256; doi:10.1371/journal.pone.0313583)
Supplement: S4 Table — (DOCX) [file pone.0313583.s004.docx]

**S4 Table: Association between LDL-C and BMD in different disease populations.**

| **Disease** | **Beta1(95%CI)** | **P Value** | **Beta2(95%CI)** | **P Value** |
| --- | --- | --- | --- | --- |
| Diabetes (2349) |  |  |  |  |
| Yes (729) | -10.9319(-19.0744, -2.7893) | 0.0101 | -5.5285(-13.5899,2.5329) | 0.1718 |
| No (1620) | -3.4134(-9.5179,2.6911) | 0.2628 | -3.5324(-9.3627,2.2978) | 0.2259 |
| Hypertension (3189) |  |  |  |  |
| Yes (1659) | -1.0369(-7.5174,5.4435) | 0.7466 | -1.6942(-7.1832,3.7949) | 0.5340 |
| No (1530) | -7.9945(-13.2323, -2.7568) | 0.0039 | -6.9412(-12.9872, -0.8952) | 0.0258 |

Abbreviation: Beta1 is the estimated coefficients of LDL-C and Spine BMD, Beta2 is the estimated coefficients of LDL-C and Femoral BMD. The analysis was conducted using a weighted logistic regression model and adjusted age, gender, race, education, PIR, BMI, HDL-Cholesterol (1-SD), LDL-Cholesterol (1-SD),Total Cholesterol (1-SD), Triglyceride (1-SD), Aspartate Aminotransferase (AST) (1-SD), Alanine Aminotransferase (ALT) (1-SD), Serum Creatinine (1-SD), Blood Urea Nitrogen (1-SD), 25-hydroxyvitamin D (1-SD), and HbA1c (1-SD), Alcoholic use, smoking status, supplements of calcium and vitamin D.
