# Supplementary figures and images for: Advantages of statin usage in preventing fractures for men over 50 in the United States: National Health and Nutrition Examination Survey
Source: PLoS One. 2024 Nov 25;19(11):e0313583. doi: 10.1371/journal.pone.0313583 (PMC11588256; doi:10.1371/journal.pone.0313583)

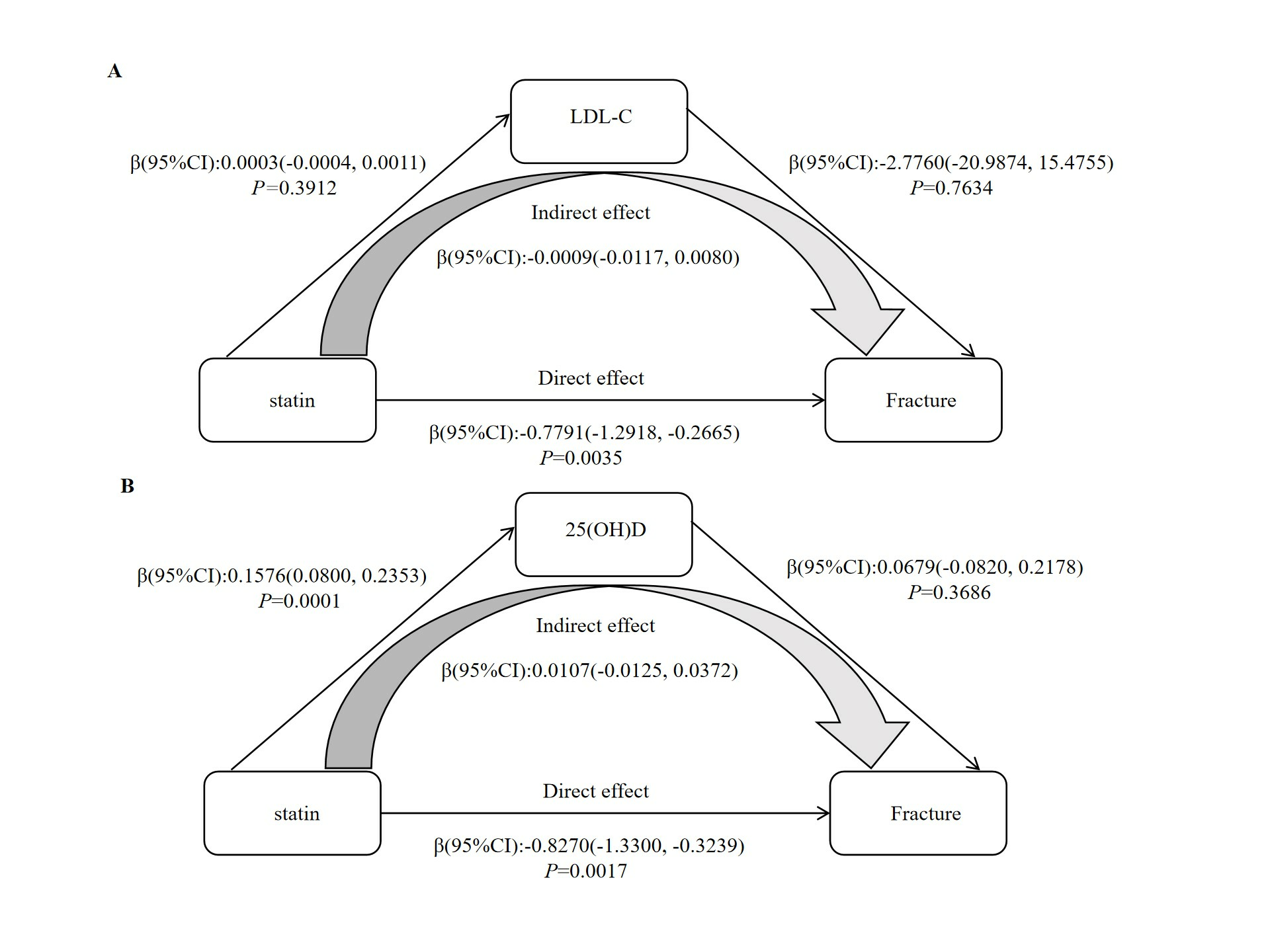

Supplement: S1 Fig — Adjusted for age, gender, race, education, PIR, BMI, Total Cholesterol (1-SD), HDL- Cholesterol (1-SD), Triglyceride (1-SD), Aspartate Aminotransferase (AST), Alanine Aminotransferase (ALT) (1-SD), Serum Creatinine (1-SD), Blood Urea Nitrogen (1-SD), and HbA1c (1-SD), smoking and drinking status, supplements of calcium and vitamin D; CI, confidence interval. (TIF) [file pone.0313583.s005.tif]
